# Heat shock protein 70 down-regulates the production of toll-like receptor-induced pro-inflammatory cytokines by a heat shock factor-1/constitutive heat shock element-binding factor-dependent mechanism

**DOI:** 10.1186/1476-9255-11-19

**Published:** 2014-07-12

**Authors:** Eduardo Ferat-Osorio, Aldair Sánchez-Anaya, Mireille Gutiérrez-Mendoza, Ilka Boscó-Gárate, Isabel Wong-Baeza, Rodolfo Pastelin-Palacios, Gustavo Pedraza-Alva, Laura C Bonifaz, Pedro Cortés-Reynosa, Eduardo Pérez-Salazar, Lourdes Arriaga-Pizano, Constantino López-Macías, Yvonne Rosenstein, Armando Isibasi

**Affiliations:** 1 Unidad de Investigación Médica en Inmunoquímica, Hospital de Especialidades, Centro Médico Nacional Siglo XXI, Instituto Mexicano del Seguro Social, Av. Cuauhtémoc 330, Col. Doctores, México D.F. CP 06020, México; 2 Servicio de Cirugía Gastrointestinal, Hospital de Especialidades, Centro Médico Nacional Siglo XXI, Instituto Mexicano del Seguro Social, Av. Cuauhtémoc 330, Col. Doctores, México D.F. CP 06020, México; 3 Departamento de Inmunología, Instituto Politécnico Nacional, Escuela Nacional de Ciencias Biológicas, México D.F., México; 4 Departamento de Biología Celular, (CINVESTAV) Instituto Politécnico Nacional, Centro de Investigación y Estudios Avanzados, México D.F., México; 5 Departamento de Medicina Molecular y Bioprocesos, Instituto de Biotecnología, Universidad Nacional Autónoma de México, Cuernavaca, México; 6 Facultad de Química, Universidad Nacional Autónoma de México (UNAM), México D.F., México; 7 Instituto de Biotecnología, Universidad Nacional Autónoma de México, Av. Universidad 2001, Col. Chamilpa, Cuernavaca Mor. 62210, México; 8 Coordinación de Investigación en Salud, Piso 4 Bloque B Unidad de Congresos Centro Médico Nacional Siglo XXI, Av. Cuauhtémoc 330, Col. Doctores, México D.F. CP 06020, México

**Keywords:** Heat shock protein 70, Inflammatory response, HSF-1, CHBF, Toll-like receptors

## Abstract

**Background:**

Heat shock protein 70 (Hsp70) is an intracellular chaperone protein with regulatory and cytoprotective functions. Hsp70 can also be found in the extracellular milieu, as a result of active secretion or passive release from damaged cells. The role of extracellular Hsp70 is not fully understood. Some studies report that it activates monocytes, macrophages and dendritic cells through innate immune receptors (such as Toll-like receptors, TLRs), while others report that Hsp70 is a negative regulator of the inflammatory response. In order to address this apparent inconsistency, in this study we evaluated the response of human monocytes to a highly purified recombinant Hsp70.

**Methods:**

Human peripheral blood monocytes were stimulated with Hsp70, alone or in combination with TLR agonists. Cytokines were quantified in culture supernatants, their mRNAs were measured by RT-PCR, and the binding of transcription factors was evaluated by electrophoretic mobility shift assay (EMSA). Kruskal-Wallis test or one-way or two-way ANOVA were used to analyze the data.

**Results:**

The addition of Hsp70 to TLR-activated monocytes down-regulated TNF-α as well as IL-6 levels. This effect was independent of a physical interaction between Hsp70 and TLR agonists; instead it resulted of changes at the TNF-α gene expression level. The decrease in TNF-α expression correlated with the binding of HSF-1 (heat shock transcription factor 1, a transcription factor activated in response to Hsp70) and CHBF (constitutive HSE-binding factor) to the TNF-α gene promoter.

**Conclusion:**

Extracellular Hsp70 negatively regulates the production of pro-inflammatory cytokines of monocytes exposed to TLR agonists and contributes to dampen the inflammatory response.

## Background

Inflammation occurs in response to infection, heat shock or cellular stress. During infection, increased levels of Heat shock protein 70 (Hsp70) confer cytoprotection [1] by inhibiting components of inflammatory signaling pathways, such as the NF-κB transcription factor [2]. Hsp70 is predominantly an intracellular protein, but it can be released to the extracellular milieu as a result of tissue damage or cellular necrosis [3]. Moreover, the presence of extracellular Hsp70 in the absence of cell death, suggests that live cells actively secrete Hsp70 [4]. It has been proposed that Hsp70 functions as an alarmin [5], as it can be detected in the plasma of healthy individuals and at higher concentrations in the serum of severely traumatized patients, patients with autoimmune diseases and inflammatory conditions, and children with septic shock [6-9]. The transcription of Hsp70 is mediated by the activation of heat shock transcription factor-1 (HSF-1), which binds to the heat shock element (HSE) in the Hsp70 gene promoter [10]. Interestingly, HSF-1 is a negative regulator of TNF-α release in a mouse model of lipopolysaccharide (LPS)-induced shock, and it represses the transcription of mouse TNF-α and IL-1β [11]. The identification of a functional HSE in the human TNF-α promoter suggests that HSF-1 directly participates in the regulation of TNF-α expression [12,13].

Increased levels of extracellular Hsp70 have been reported to stimulate an inflammatory response via a Toll-like receptor 2 (TLR2)/TLR4/CD14-dependent mechanism that leads to NF-κB activation, and TNF-α, IL1-β and IL-6 production [14]. However, other reports show that extracellular Hsp70 induces LPS tolerance and prevents the augmentation of pro-inflammatory cytokines levels that follows LPS stimulation [15]. The immune-stimulating activity of Hsp70 through TLR2 and TLR4 remains thus controversial, and it has been proposed that the pro-inflammatory activity of Hsp70 results rather of contaminating LPS [16]. Furthermore, a pro-inflammatory role of extracellular Hsp70 would be difficult to reconcile with the role of intracellular Hsp70, which mainly regulates apoptosis and confers cytoprotection [1,17]. In fact, data from the HSP70 1/3 knockout mice suggests that extracellular Hsp70 is important for the negative regulation of inflammatory mediators during systemic infection [18].

In this study, we evaluated the role of extracellular Hsp70 on the inflammatory response elicited by different TLR agonists central to the early onset of innate immune responses to bacterial infections.

## Methods

### Reagents

Recombinant human stress induced Hsp70 (Hsp72) was purchased from Enzo Life Sciences (formerly Assay Designs, and formerly Stressgen Biotechnologies Corporation). We tested conventional, non-LPS-free Hsp70 (NSP-555), which we will refer to as “HE/Hsp70”; and LPS-free Hsp70 (ESP-555), which we will refer to as “Hsp70”. The non-LPS free HE/Hsp70 contains 200 pg of LPS per μg of protein, while the LPS-free Hsp70 contains 1.4 pg of LPS per μg of protein (fewer than 50 IU of LPS), as assessed by the *Limulus* amebocyte lysate assay in our laboratory. According to the manufacturer, both Hsp70preparations retain their ATPase activity. To heat-denature Hsp70, the protein was boiled for 120 min. *Escherichia coli* 0111:B4 LPS was from Sigma Chemical Co. (St. Louis, MO, USA); *Salmonella typhimurium* flagellin and *Staphylococcus aureus* peptidoglycan were from Invivogen (San Diego, CA, USA), and vaccine-grade *Salmonella enterica* serovar *Typhi* porins, with < 200 pg of LPS per μg of protein, were obtained in our laboratory as previously reported [19].

### Cell separation and stimulation

Peripheral blood mononuclear cells from leukocyte concentrates of healthy blood bank donors were isolated with Lymphoprep™ (Axis-Shield, Oslo, Norway). Mononuclear cells were resuspended in 2 ml of RPMI 1640 supplemented with 10% fetal bovine serum and 1% penicillin-streptomycin (all reagents were from GIBCO Invitrogen, Carlsbad, CA, USA), counted and plated at 20 × 10^6^ cells per 15 ml in a culture dish for 2 h at 37°C. The monocytes-enriched fraction was detached with cold buffer (NaCl 0.8%, KCl 0.04%, glucose 0.1%, sodium EDTA 0.02% and NaHCO_3_ 0.6%) and plated at 1 × 10^6^ cells per well in 24-well plates for 24 h at 37°C and 5% CO_2_ in supplemented RPMI. All experiments were performed with mononuclear cells enriched for monocytes by at least 85% as assessed by flow cytometry expression.

After 24 h, the culture medium was removed and cells were washed twice with phosphate-buffered saline before adding fresh supplemented RPMI with or without LPS (100 ng/ml), peptidoglycan (10 μg/ml), flagellin (10 μg/ml) or porins (3 μg/ml). The TLR agonists were added alone or in the presence of the indicated amounts of Hsp70 in 1 ml of RPMI, for the indicated times. Supernatants were then collected, centrifuged (3,000 rpm for 5 min), and frozen at −70°C. Alternatively, TLR agonists were pre-incubated with Hsp70 in 200 μl of binding buffer (25 mM TRIS, 20 mM HEPES, 47.5 mM KCl, and 2.25 mM Mg(OAc)_2_, pH 7.15) for two hours at 37°C, before adding them to the cells.

### Cell separation and stimulation for flow cytometric analysis of TLR expression

Peripheral blood mononuclear cells were obtained from leukocyte concentrates of healthy blood-bank donors by gradient centrifugation with Lymphoprep™ (Axis-Shield, Oslo, Norway). Mononuclear cells were resuspended in 2 ml of RPMI 1640 supplemented with 10% fetal bovine serum and 1% penicillin-streptomycin (all reagents from GIBCO Invitrogen, Carlsbad, CA, USA), counted and seeded at 0.5 × 10^6^ cells per tube and incubated in a humidified incubator with 5% CO_2_ and 37°C.

### Cytokine quantification

The concentrations of TNF-α, IL-6 and IL-10 in cell culture supernatants were measured with ELISA kits (BD Biosciences Pharmingen, San Jose, CA, USA), according to the manufacturers’ protocols.

### PCR quantification

The TNF-α mRNA was analyzed by RT-PCR. Total RNA was extracted from monocytes using TRIzol reagent (Invitrogen). The concentration and purity of RNA were determined at 260 and 280 nm. Single-stranded cDNA was synthesized by mixing 1 μg RNA with 1 μl of oligo-dT_(12–18)_ (0.5 μg/μl, Invitrogen), 1 μl of a dNTP mixture (10 mM each), 2 μl of 0.1 M dithiotreitol and 200 U of SuperScript II RNase H reverse transcriptase in a total volume of 20 μl; the reaction mixture was incubated at 42°C for 50 min, followed by 70°C for 15 min to inactivate the reverse transcriptase. cDNA (1 μg) was used as a template for PCR amplification of the TNF-α gene. The PCR reaction mixture consisted of 0.5 μl of a dNTP mixture (10 mM each), 1.5 μl of 25 mM MgCl_2_, 1 μl of primer working stock (10 pmol of each primer), 0.25 μl of dimethilsulfoxide (Sigma) and 1.25 U of *Taq* DNA polymerase in a total volume of 20 μl (all reagents were from Promega, Madison, WI, USA, unless otherwise indicated). The TNF-α primers were 5′-GGT-GCT-TGT-TCC-TCA-GCC-TC-3′ and 5′-CAG-GCA-GAA-GAG-CGT-GGT-G-3′ [20] and the β-actin primers were 5′-GTG-GGG-CGC-CCC-AGG-CAC-CA-3′ and 5′-CTC-CTT-AAT-GTC-ACG-CAC-GAT-TTC-3′ [21]. β-actin was used as an internal control in each experiment. The reaction mixture was denatured at 95°C for 10 min, followed by 35 cycles of denaturation (95°C/30 s), annealing (58°C/1 min), and extension (72°C/1 min); the final extension was for 10 min. The PCR products were separated by electrophoresis in agarose gels at 70 V for 60 min, stained with ethidium bromide and observed by ultraviolet illumination. Images were captured and analyzed with an IS-1000 Digital Imaging System (Alpha-Innotech Corporation, San Leandro, CA, USA). The pixel densities of TNF-α and β-actin bands were determined and the ratios of TNF-α to β-actin were calculated; these ratios represent the relative expression levels or expression index of TNF-α mRNA.

### Electrophoretic mobility shift assay (EMSA)

To prepare nuclear extracts, monocytes were lysed in 1 ml of cold buffer A (10 mM Tris–HCl pH 7.4, 10 mM NaCl, 6 mM MgCl_2_, 10 mM NaF, 1 mM Na_3_VO_4_, 1 mM dithiotreitol, and 1 mM phenylmethanesulfonyl fluoride) and incubated on ice for 10 min. After this incubation, 10 μl of IGEPAL 10% were added, and the tubes were incubated for another 5 min at 4ºC on a shaking platform. The lysates were centrifuged at 2,600 rpm for 5 min, resuspended in 40 μl of cold buffer B (20 mM HEPES, pH 7.9, 420 mM NaCl, 20% glycerol, 1.5 mM MgCl_2_, 0.2 mM EDTA, 1 mM Na_3_VO_4_, 10 mM NaF, 1 mM DTT, and 0.2 mM PMSF) and incubated for 15 min at 4ºC on a shaking platform. Nuclear extracts were recovered by centrifugation at 12,000 rpm for 15 min at 4°C and frozen at −70°C. Total protein concentration was measured using a commercial reagent (Bio-Rad Laboratories, Hercules, CA, USA) and a bovine serum albumin standard curve. To evaluate the binding of HSF-1 to the promoter region of the human TNF-α gene, we used a double-stranded oligonucleotide that corresponds to the +45/+73 position of the TNF-α sequence (5′-AG*A-GAA-G*CA-ACT-ACA-GAC-CCC-CCC-*TGA-AA*-3′) and contains specific binding sites for HSF-1 (in boldface). The oligonucleotide was labeled with [γ-^32^P] ATP with a T4 polynucleotide kinase (Fermentas, Thermo Scientific, Glen Burnie, MD, USA), according to the manufacturer’s protocol. The ^32^P-labelled oligonucleotide probe (1 ng) was incubated with 5 μg of nuclear extract in the presence of 3 μg poly(dI-dC) in 0.25 M HEPES, pH 7.5, 0.6 M KCl, 50 mM MgCl_2_, 1 mM EDTA, 7.5 mM DTT and 9% glycerol) for 20 min at 4°C. We added a 50-fold excess of unlabeled TNF-α probe (“cold”) or an irrelevant oligonucleotide (5′-ACG-TGT-GAT-GAA-ATG-CTA-GGC-GAT-C-3′) as specific and non-specific competitors, respectively. After incubation, samples were resolved on 6% polyacrylamide gels in 0.5x Tris borate-EDTA buffer (74.5 mM Tris–HCl, 1.6 mM sodium EDTA, and 44.5 mM boric acid pH 8.5) for 4 h at 0.28 mA. The gels were dried and analyzed by autoradiography with a Kodak intensifying screen (Carestream Health, Rochester, NY, USA) at −80°C.

### Fluorescence-activated cell sorting (FACS) analysis of TLR and CD14 expression

FACS was performed to detect cell surface expression of TLR2, TLR4 on CD14 cells after Hsp70 stimulation. Human mononuclear cells were exposed to 3 μg/ml Hsp70 for 1 or 4 hours, and then the cells were harvested and washed twice with PBS containing 1% BSA. Cells were incubated with FITC-conjugated anti-TLR2, PE-conjugated anti-TLR4 (eBioscienced) and Pacific Blue-conjugated anti-CD14 (BioLegend), at room temperature for 30 min. Fluorescence was determined by a FACScan flow cytometry (Becton-Dickinson).

### Statistical analysis

Each experiment was performed three or four times with independent donors. Data were analyzed with GraphPad Prism 5.0 software (GraphPad Software, San Diego, CA, USA). Kruskal-Wallis test, one-way or two-way ANOVA were used as required, followed by Dunn’s multiple comparison test, Tukey or Bonferroni post-tests. A P value < 0.05 was considered statistically significant.

## Results

### Hsp70 decreases the production of TNF-α by LPS-activated monocytes

**Figure 1:**
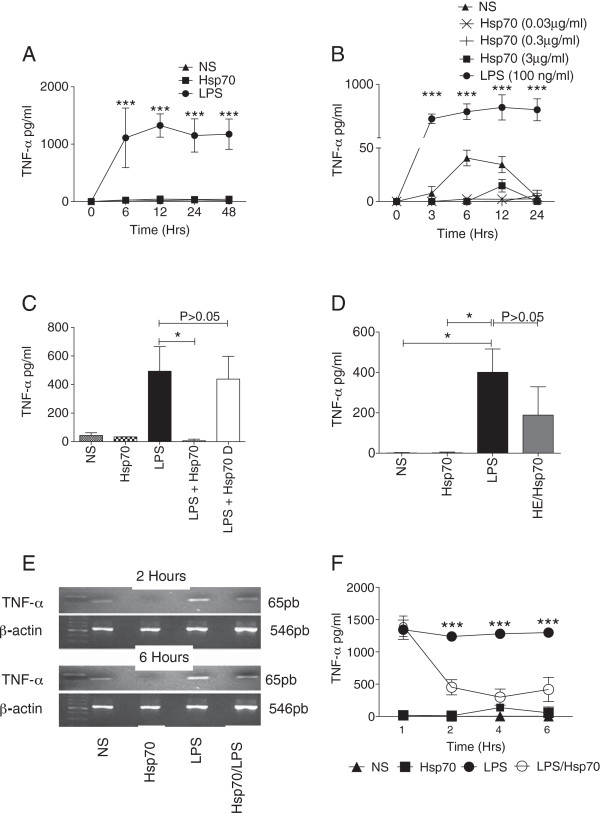
**Hsp70 decreases the production of TNF-α by LPS-activated human monocytes. A**. Monocytes were left unstimulated (NS) or stimulated with Hsp70 (3 μg/ml) or LPS (100 ng/ml) for the indicated time and TNF-α concentration was measured in the supernatants. Two-way ANOVA with Bonferroni post-test: ***P < 0.001 LPS vs. Hsp70. **B**. Monocytes were unstimulated (NS) or stimulated with different concentrations of Hsp70 (3, 0.3 and 0.003 μg/ml) or LPS (100 ng/ml) and the amount of TNF-α in the culture supernatant was measure at the indicated time. Two-way ANOVA with Bonferroni post-test: ***P < 0.001 LPS vs. Hsp70. **C**. Monocytes were non stimulated (NS) or stimulated for 6 h with LPS-purified Hsp70 (3 μg/ml), LPS (100 ng/ml), Hsp70 and LPS or LPS and heat-denatured Hsp70 (Hsp70 D) as described in material and methods. TNFα production was determined after 6 hr in the culture supernantant. Kruskal-Wallis test with Dunn’s multiple comparison test: *P < 0.05. **D**. Monocytes were non stimulated (NS) or stimulated for 6 h with LPS-purified Hsp70 (3 μg/ml), LPS (100 ng/ml) or non-LPS-purified Hsp70 (HE/Hsp70, 3 μg/ml) and TNFα production was determined after 6 hr in the culture supernantant. Kruskal-Wallis test with Dunn’s multiple comparison test: *P < 0.05. **E**. Monocytes were cultured non treated (NS) or cultured with LPS (100 ng/ml) in the presence or absence of Hsp70 (3 μg/ml) for 2 or 6 hrs, and the expression levels of TNF-α mRNA were evaluated. β-actin was amplified simultaneously to verify RNA integrity and to ensure that equivalent amounts of templates were used. **F**. TNF-α production by the monocytes in **E** at four different times 1, 2, 4 and 6 hrs. Kruskal-Wallis test with Dunn’s multiple comparison test: *P < 0.05. NS = non-stimulated cells. Data is representative of 3 independent assays.

Hsp70 is released by cell injury secondary to infections [7], and some authors have proposed that extracellular Hsp70 is a TLR2 and/or TLR4 agonist that induces the production of pro-inflammatory cytokines [22], while other authors argue that an LPS contamination of the Hsp70 used in these experiments is accountable for the inflammatory response [16,23]. To re-examine these possibilities, we evaluated the capacity of LPS-free Hsp70 to trigger the production of the pro-inflammatory cytokine TNF-α by human monocytes. We confirmed that extracellular Hsp70 signals through TLR2 and TLR4 as it induced the recruitment of NF-κB to DNA (Additional file 1: Figure S1). However stimulation with Hsp70 (3 μg/ml) for different times (0, 3, 6,12, 24 and 48 h) as well as with concentrations ranging from 0.03 to 3 μg/ml did not induce TNF-α production (Figure 1A and B). Interestingly, TNF-α concentration was lower when monocytes were exposed to LPS-free Hsp70 in comparison with non-stimulated monocytes (Figure 1B); although this difference was not statistically significant, it was consistent through all experiments. In contrast, non-LPS-free HE/Hsp70 clearly induced the production of TNF-α (Figure 1D).

Hsp70 has been reported to inhibit some components of the pro-inflammatory signaling cascades [2]. To investigate this possibility, we added LPS-free Hsp70 and LPS to the same well, and found that Hsp70 considerably decreased the amount of TNF-α secreted by monocytes in response to LPS. This effect was lost if the Hsp70 was heat-denatured before the assay (Figure 1C). To assess if Hsp70 prevented LPS-induced TNF-α transcription, we examined the levels of TNF-α mRNA in LPS-activated monocytes. Hsp70 reduced the TNF-α mRNA levels resulting of a 2 or 6 hr LPS stimulation (Figure 1E) with similar results at 1, and 4 h after LPS activation (data not shown). The reduced mRNA levels correlated with lower levels of TNF-α in the supernatants (Figure 1F). Hsp70 had no effect on the surface expression of TLR2 and TLR4 (Additional file 2: Figure S2).

Altogether this data support the hypothesis that LPS-free Hsp70 is not a pro-inflammatory signal for the cell, and furthermore that it dampens TNF-α production.

### Hsp70 regulates LPS-dependent TNF-α production through a mechanism that does not depend on the direct interaction between LPS and Hsp70

**Figure 2:**
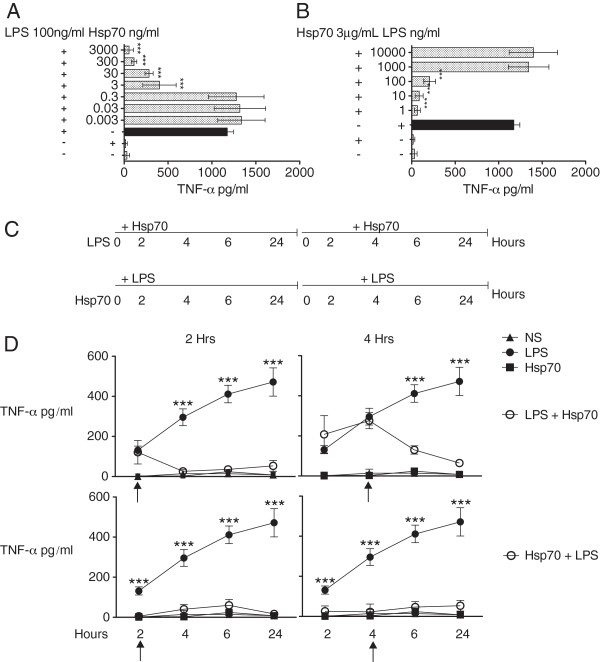
**Hsp70 regulates LPS-dependent TNF-α production through a mechanism that does not depend on the interaction between LPS and Hsp70. A**. Monocytes were stimulated for 6 h in the presence of Hsp70 (3000 to 0.003 ng/ml) and 100 ng/ml of LPS. **B**. Monocytes were stimulated for 6 h in the presence of LPS (10000 to 1 ng/ml) and 3 μg/ml of Hsp70. LPS was pre-incubated with Hsp70 in 200 μl of binding buffer for 2 h at 37°C, before adding them to the corresponding wells. TNF-α production was determined after 6 hr in the culture supernantant. One-way ANOVA with Tukey’s post-test: ***P < 0.001 LPS vs. LPS + Hsp70. Data is representative of 3 independent assays. **C**. Schematic representation of the experimental design. **D**, top panels. Monocytes were cultured in the absence (NS) or in the presence of LPS (100 ng/ml) for 2 or 4 h. After this incubation, fresh culture medium with Hsp70 (3 μg/ml) was added (LPS + HSp70). The cells were incubated for 24 h. As controls, LPS (100 ng/ml) or Hsp70 (3 μg/ml) were added to the cells. The amount of TNF-α was measured in the supernatants at the indicated time points. **D**, bottom panels. Monocytes were left unstimulates (NS) or cultured with Hsp70 (3 μg/ml) for 2 or 4 h. After this incubation, fresh culture medium with LPS (100 ng/ml) was added (Hsp70 + LPS). As controls, LPS (100 ng/ml) or Hsp70 (3 μg/ml) were added to the cells. The cells were incubated for a total of 24 h. The amount of TNF-α was measured in the supernatants at the indicated time periods. Two-way ANOVA with Bonferroni post-test: ***P < 0.001 LPS vs. Hsp70 + LPS or vs. LPS + Hsp70. NS = non-stimulated cells. Data is representative of 3 independent assays.

**Figure 3:**
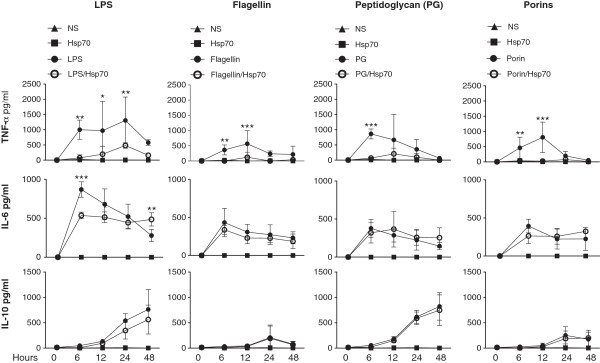
**Hsp70 decreases the production of TNF-α and IL-6, but not that of IL-10, in response to TLR agonists.** Monocytes were left untreated (NS) or cultured for 48 h in the presence of LPS (100 ng/ml), flagellin (10 μg/ml), peptidoglycan (PG) (10 μg/ml) and porins (3 μg/ml), alone or in the presence of Hsp70 (3 μg/ml). TLR agonists were pre-incubated with Hsp70 in 200 μl of binding buffer for 2 h at 37°C, before adding them to the corresponding wells. The concentrations of TNF-α, IL-6 and IL-10 were measured in the supernatants at the indicated time periods. Two-way ANOVA with Bonferroni post-test: *P < 0.05; **P < 0.01; ***P < 0.001 each TLR agonist vs. each TLR agonist + Hsp70. NS = non-stimulated cells. Data is representative of 3 independent assays.

In order to further characterize the effect of Hsp70, we first performed a checkerboard assay on monocytes incubated with different amounts of LPS and LPS-free Hsp70 and measured TNF-α production. The inhibitory effect of HSP-70 on TNF-α production is dose-dependent. As little as 3 ng/ml of Hsp70 significantly reduced the amount of TNF-α produced in response to 100 ng/ml of LPS (Figure 2A) and 3 μg/ml abolished the TNF-α production in response to 100 ng/ml of LPS by 34%, but not that in response to 1000 or 10000 ng/ml of LPS (Figure 2B).The ability of Hsp70 to reduce the amount of TNF-α produced in response to LPS could result of the physical association between LPS and Hsp70, potentially interfering with TLR4 signaling. To test this possibility, we used a “two-step” stimulation model (Figure 2C). Monocytes were first stimulated with LPS or Hsp70 for 2 or 4 h, after which, the wells were extensively washed with culture medium and fresh culture medium with Hsp70 or LPS was added. In the upper left panel of Figure 2D, the “TNF-α values shown at 2/4 hours for the LPS + Hsp70 and Hsp70 samples represent the amount of TNF-α present in those wells at the time where the Hsp70 was additioned to the cells”. Therefore, as expected, there is no significative difference between the TNF-α values in the LPS wells and the LPS-Hsp70 wells at the 2 hr or 4 hr point for the right upper panel of Figure 2D. These values shaw the “evolution” of TNF-α release prior to Hsp70 addition, as all cells were seeded at the same time (Figure 2D, top panels). The TNF-α values shown at 2/4 hours for the LPS + Hsp70 and Hsp70 samples represent the amount of TNF-α present in the wells at the time where the Hsp70 was additioned to the cells. Likewise, addition of LPS did not rescue TNF-α production when added after Hsp70, even though Hsp70 was no longer present (Figure 2D, bottom panels).

These data suggests that, depending on the concentration, Hsp70 can override LPS-mediated signals, and that this effect does not depend on the ability of Hsp70 to directly interact with LPS.

### Hsp70 decreases the production of TNF-α, but not that of IL-10, in response to TLR agonists

To assess whether the capacity of Hsp70 to inhibit TNF-α production was restricted to LPS, we sought to stimulate human monocytes with other TLR agonists: *Staphylococcus aureus* peptidoglycan, a TLR2 agonist; *Salmonella enterica* serovar *Typhi* porins, TLR2 and TLR4 agonists [24]; *Escherichia coli* LPS, a TLR4 agonist and, *Salmonella typhimurium* flagellin, a TLR5 agonist. The addition of Hsp70 clearly lowered the production of TNF-α by monocytes in response to all TLR agonists. Hsp70 also reduced the amount of the pro-inflammatory cytokine IL-6 released in response to LPS but interestingly had no effect on IL-6 production in response to flagellin, peptidoglycan or porins. In contrast, the addition of Hsp70 did not impact the levels of the anti-inflammatory cytokine IL-10 secreted in response to LPS or any other TLR agonists (Figure 3). These results show that Hsp70 selectively affects the production of pro-inflammatory cytokines such as TNF-α, but not that of anti-inflammatory ones such as IL-10.

### Hsp70-induced binding of HSF-1 to the TNF-α promoter correlates with decreased TNF-α protein levels

**Figure 4:**
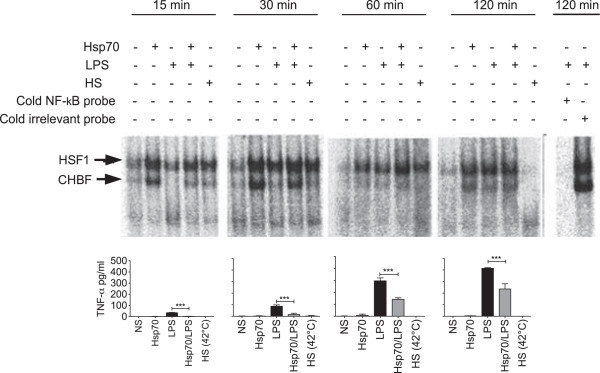
**The effect of Hsp70 on LPS-induced TNF-α production depends on the binding of HSF-1 and CHBF to the TNF-α promoter.** Top panels. Monocytes were stimulated with Hsp70 (3 μg/ml), LPS (100 ng/ml) or Hsp70 + LPS for 15, 30, 60 and 120 min, at 37°C. Non-stimulated cells were used as a negative control (−) and monocytes cultured at 42°C for the indicated times (heat-shock, HS) were used as a positive control. After incubation, nuclear extracts were obtained and analyzed by EMSA, using the TNF-α probe. The autoradiography is representative of 3 independent experiments. The slower migrating complex, corresponds to the binding of HSF-1 to the HSE in the TNF-α promoter, and the faster migrating complex, corresponds to the binding of CHBF to the HSE. Control included EMSA reaction with 50-fold excess cold TNF-α competitor or 50-fold excess irrelevant competitor. Bottom panels. The levels of TNF-α were determined in the supernatant of the same cultures; data is from 3 independent experiments. Two-way ANOVA with Bonferroni post-test: ***P < 0.001.

The mouse TNF-α promoter contains a binding site for HSF-1, and binding of HSF-1 to its HSE results in repressed transcription [12]. To test if the decrease in TNF-α production we detected with Hsp70 in LPS-activated human monocytes involved HSF-1 recruitment, we evaluated the interaction of this transcription factor with the promoter region of the human TNF-α gene by EMSA (Figure 4). Consistent with the literature, two distinct HSE-binding complexes were identified: a slower-migrating complex corresponding to the binding of HSF-1 to the HSE in the TNF-α gene promoter, and a faster-migrating complex corresponding to the binding of CHBF (constitutive HSE-binding factor, also known as Ku autoantigen) to HSE [25,26]. We found that HSF-1 as well as CHBF constitutively bound to the promoter region of the TNF-α gene in resting cells, but that they were highly enriched (4-fold approximately) in Hsp70-activated cells with both proteins recruited in equivalent amounts. Co-stimulation with LPS and Hsp70 favored the formation of the faster migrating complex (CHBF binding) in addition to the slower migrating complex (HSF-1 binding). Stimulating the cells with LPS resulted in the recruitment of HSF-1 only and the faster migrating complex appears 60 min following stimulation, albeit in a smaller proportion as compared to HSP70- or HSP70/LPS-stimulated cells (Figure 4, top panels). The specificity of the interaction of HSF-1 with the TNF-α promoter was demonstrated by inhibition of the formation of the HSF-1 complex in the presence of a 50-fold excess of a “cold” competitor, while an irrelevant competitor had no effect on the binding. As expected, a heat shock (performed by incubating the monocytes at 42°C for the indicated times) also induced the binding of HSF-1 to the TNF-α promoter (Additional file 3: Figure S3). The decrease in TNF-α concentration in the supernatants in response to LPS + Hsp70, compared to the amount of TNF-α in response to LPS alone, correlates with the increased binding of HSF-1 to the TNF-α promoter in response to LPS + Hsp70 (Figure 4, bottom panels). Altogether, these data suggest that Hsp70 negatively controls TNF-α gene transcription by recruiting HSF-1 and CHBF to the TNF-α gene promoter.

## Discussion

An increasing number of diseases result of a dysregulated inflammatory response, and several inflammatory mediators, including Hsp70, have been implicated. The molecular chaperone Hsp70 has many functions depending on the cell type, and our understanding of the roles it may play, especially the extracellular form, remains incomplete, and is even controversial [27-32]. Two members of the HSP70 family, Hsp73 and Hsp72, share a high degree of sequence homology but differ in their expression pattern: Hsp73 is constitutively expressed, while Hsp72 is stress-inducible [33]. In resting cells, Hsp73 is found mainly in the cytoplasm, the nuclei and nucleoli, while Hsp72 is found in nuclei and nucleoli. However, after a stressful event, such as heat shock, the distribution of both forms is homogenized throughout the cell, suggesting that both isoforms associate with similar molecules to protect essential cell structures [34]. Incubation of human monocytes with Hsp70 –the inducible form- was reported to have a functional consequence by eliciting the rapid expression of pro-inflammatory cytokines [14]; whereas Hsp73 does not induce any significant release of IL-6 – a pro-inflammatory cytokine by monocytes [35].

Some authors have reported that extracellular Hsp70 functions as a DAMP and activates monocytes, macrophages and dendritic cells, potentially through cell surface receptors such as CD14, CD40, CD91, Lox1, TLR2 and TLR4 [36]. In different experimental models [14,22,37-39], Hsp70 has been found to induce intracellular calcium fluxes and NF-κB activation, with the subsequent production of the pro-inflammatory cytokines TNF-α, IL-1β, IL-6 and IL-8. However, Hsp70 is present in the peripheral circulation of healthy subjects at concentrations that, according to these studies, would elicit cytokine production [8], suggesting that Hsp70 is not pro-inflammatory in all contexts [40,41]. In fact, intracellular Hsp70 has a cytoprotective effect, as it inhibits the production of inflammatory mediators during cellular stress [42]. Our results support a cytoprotective role for Hsp70 whereby it diminishes the production of the pro-inflammatory mediator TNF-α, through the recruitment of HSF-1 transcription factor to the cytokine promoter, thus extending its cytoprotective role beyond the intracellular space to the extracellular milieu.

Data shown here bring also evidence that extracellular Hsp70 not only fails to induce TNF-α production in human monocytes, but that it down-regulates their capacity to produce TNF-α and IL-6 in response to LPS. The Hsp70 (LPS-free HSP70) we used in our experiments corresponds to the inducible isoform (Hsp72); as a result of a multi-step chromatography purification process it has very low levels of LPS (<50 endotoxin units/mg) and could not, by itself, induce cytokine production by human monocytes. These results are consistent with reports that attribute the immune-stimulating activity of Hsp70 to contaminating LPS [16,43], and others that show that in patients with rheumatoid arthritis, extracellular Hsp70 decreased IL-6, IL-8 and MCP-1 production induced by TNF-α [44]. Likewise, recombinant Hsp72 (induced Hsp70) has been reported to lessen the severity of collagen-induced arthritis in mice, by lowering serum TNF-α and IL-6 concentration [45].

We used a highly purified commercial Hsp70 for our experiments (purified by multi-step chromatography, with <50 endotoxin units/mg); this Hsp70 corresponds to the inducible isoform (Hsp72), and it is not bound to any other molecule. However, we cannot exclude the possibility that Hsp70 bound another protein or peptide in the cell culture supernatants. This binding, as well as its ADP/ATP-binding status, could modify the effects of Hsp70 on human monocytes [46]. These two aspects need to be evaluated in future studies. The recombinant Hsp70 that we used is produced in *E. coli*, so it is possible that its conformation is different from that of native Hsp70 in cells. However, this Hsp70 from Enzo Life Sciences, formerly Stressgen retains its ATPase activity, and we observed that it activates NF-κB through TLR2 and TLR4 signalling, as has been reported previously which suggest that the recombinant protein retains a conformation compatible with its biological activity [16,47].

Hsp70 interacts with highly hydrophobic peptides [48]. TLR agonists usually have hydrophobic regions that bind to the leucine-rich-repeats (LRR) domain-groove of TLRs [49]. The binding of a TLR agonist to Hsp70 could interfere with its ability to engage its TLR, and the subsequent signaling cascade leading to cytokine production would be defective. Our results do not support this possibility. Data we report here suggest rather that the effect of Hsp70 on LPS-induced TNF-α production is independent of a physical association with LPS, because the addition of LPS after monocytes were treated with Hsp70 did not lead to TNF-α production, even though Hsp70 was no longer present in the system. Furthermore, our results indicating that Hsp70 activates NF-κB through TLR2 and TLR4 signaling argues against the possibility that Hsp70 inhibit LPS-mediate TNF-α expression by negatively modulating TLR4 expression. In fact, we found that Hsp70 has no effect on the surface expression of TLR2 and TLR4 on monocytes, consistent with a report showing that Hsp70 does not affect TLR2 nor TLR4 expression on mouse macrophages [50].

TNF-α production by human peripheral blood mononuclear cells was reduced by more than 50% when the cells were cultured with LPS in the presence of Hsp70, but that incubating these cells with 15 to 60 μg/ml of Hsp70 for 24 h increased TNF-α production in response to 1 ng/ml of LPS [43]. This apparent discrepancy with our results could be caused by the different concentrations of Hsp70 and LPS used, and by the use of peripheral blood mononuclear cells instead of enriched monocytes for the experiments.

Endotoxemia stimulates stress responses through HSF-1, as a protective mechanism for the host. HSF-1 is found in the cytoplasm as a monomer devoid of transcriptional activity, but upon exposure to a heat shock or to other stressing insults, it is phosphorylated, trimerized and transported to the nucleus, where it binds to HSEs. Extracellular Hsp70 phosphorylates Akt through the TLR4 signaling pathway, resulting in glycogen synthase kinase (GSK)-3β inactivation [51]. The inactivation of GSK-3β, an inhibitory regulator of HSF-1, leads to increased HSF-1 activity [52]. CHBF interacts also with HSE and competes with HSF1 for binding to DNA [53]. The mouse TNF-α promoter contains an HSE binding site for HSF-1 and binding of HSF-1 this HSE results in repressed transcription [12]. Based on the location of the putative HSE in the human TNF-α gene promoter, HSF-1 and CHBF could hinder the RNA polymerase processivity [12]. In our experimental system, we provide evidence that the decrease in TNF-α production caused by Hsp70 in LPS-activated monocytes results of a change at the gene expression level: the amount of TNF-α mRNA was decreased in the presence of Hsp70. We report that monocyte co-stimulation with LPS and Hsp70 lead to HSF-1 and CHBF binding to the HSE on the TNF-α promoter, and that this binding correlated with a decreased production of TNF-α. As expected, unstressed cells contained mainly the slower migrating complex. These data are consistent with the fact that heat shock, which increases the levels of HSF-1, has a suppressive effect on the inflammatory response, possibly because HSF-1 exerts a competitive inhibition on NF-κB binding [54]. We observed that Hsp70 activates NF-κB through TLR2 and TLR4 signaling (Additional file 1: Figure S1). However, the activation of this transcription factor did not lead to TNF-α production, probably as a result of the strong activation of HSF-1. The reduction of IL-6 production in our model could be explained because HSF-1 inhibits the expression of this cytokine through activating transcription factor 3 (ATF-3) [55].

Interestingly, we found that Hsp70 treatment prevented LPS-induced IL-6 expression but not that resulting from engaging TL2 (porins) o TLR5 (flagellin). Unlike TLR2 and TLR5, TLR4-induced IL-6 expression involves the activation of the JAK-STAT pathway, thus making IL-6 expression susceptible to the negative regulation of SOCS1 [56]. Noteworthy, *T. gondii* Hsp70 promotes SOCS1 expression in macrophages [57]. Thus, it is possible that by inducing SOCS1 expression H70 specifically preventsTRL-4 induced JAK-STAT-dependent IL-6 expression, without affecting that resulting from TLR2 or TLR5 signaling.

A previous paper reported that exogenous recombinant Hsp70 interacts with HSF-1 in cell lines. The authors find that Hsp70 binds to HSF-1 and blocks its DNA-binding activity, and they suggest a model in which this association prevents the transcription of HSF-1-regulated genes in resting cells. After a heat shock, miss-folded proteins compete for Hsp70 binding, and this releases HSF-1 [58]. In our study, we did not explore the signalling pathways that lead to HSF-1 activation, but a similar mechanism could operate during inflammation-induced cell stress. Other studies report that HSF-1 deficient mice have increased levels of TNF-α after stimulation with LPS [59], and that *Escherichia coli*-induced inflammation is resolved faster when a heat shock precedes inoculation with bacteria [60]. HSF-1 also inhibits the transcription and secretion of G-CSF in response to LPS [61]. Furthermore, HSF-1 has been reported to prevent the overproduction of pro-inflammatory cytokines in conditions such as sepsis [62], potentially decreasing the inflammation-associated damage. Our results support the idea that exogenous HSP70 provides a mechanism for controlling the excessive expansion of an inflammatory response after monocytes activation by bacterial pathogens through the recruitment of HSF-1 to the human TNF-α promoter, and underscores a potential role for exogenous Hsp70 as a prophylactic agent for different types of inflammatory diseases associated with infections.

## Conclusion

Extracellular Hsp70 negatively regulates the production of pro-inflammatory cytokines of monocytes exposed to TLR agonists and contributes to dampen the inflammatory response.

## Abbreviations

Hsp70: Heat shock protein 70; TLR: Toll-like receptors; RT-PCR: Reverse transcription polymerase chain reaction; EMSA: Electrophoretic mobility shift assay; TNF-α: Tumor necrosis factor alfa; IL-6: Interleukine 6; IL-10: Interleukine 10; NF-κB: Nuclear factor kappa B; HSF-1: Heat shock transcription factor 1; HSE: Heat shock element; CHBF: Constitutive HSE-binding factor.

## Competing interests

The authors declare that they have no competing interests.

## Authors’ contributions

EFO, YR, CLM and AI conceived the study and designed the experiments; EFO, ASA, MGM, IBG, IWB, RPP and LAP performed experiments and/or participated in data analysis and interpretation; GPA, PCR and EPS contributed to the design of the EMSA and the collection of data; EFO, IWB, GPA and YR wrote the manuscript. All authors read and approved the final manuscript.

## Supplementary Material

Additional file 1: Figure S1 Hsp70 activates NF-κB through TLR2 and TLR4 signaling. Peripheral blood mononuclear cells were blocked with 5 μg of anti-TLR2 antibody (iTLR2) and/or 30 μg of anti-TLR4 antibody (iTLR4). After 30 min, cells were washed and stimulated with 3 μg of Hsp70, 100 ng of LPS or 10 μg of peptidoglycan (PGN) for 120 min. After this incubation, nuclear extracts were obtained and analyzed by EMSA. The EMSA was performed as indicated in Material and Methods, using a double-stranded oligonucleotide that contains specific binding sites for NF-│B (5′–AGC-TAA-GGG-ACT-TTC-CGC-TGG-GGA-CTT-TCC-AGG–3′). Control included EMSA reaction with 50-fold excess cold NF-│B competitor or 50-fold excess irrelevant competitor. The autoradiography is representative of two independent experiments. The NF-κB complex is indicated (arrow). Click here for file

Additional file 2: Figure S2 Hsp70 has no effect on the surface expression of TLR2 and TLR4 on monocytes. Human peripheral blood mononuclear cells (5 × 10^5^) were stimulated with 3 μg/ml Hsp70 or 100 ng/ml of LPS (as a positive control of cellular activation) for 1 (a-b) or 4 hours (c-d). Surface expression of TLR2 (a-c) and TLR4 (b-d) in CD14+ cells was analyzed by flow cytometry after staining the cells with FITC-conjugated anti-TLR2, PE-conjugated anti-TLR4 and Pacific Blue-conjugated anti-CD14 (BioLegend) at room temperature for 30 min. TLR expression was normalized and is shown as fold expression, relative to untreated cells (NS). Data represent the mean ± SD of three independent experiments. Click here for file

Additional file 3: Figure S3 Heat-shock induces HSF-1 binding to the human Hsp70 promoter. Peripheral blood mononuclear cells were incubated at 42°C for 1 h (heat-shock, HS), or stimulated with 100 ng/ml of LPS and incubated at 37°C for 1 h and nuclear extracts were obtained and analyzed by EMSA. The EMSA was performed as indicated in Material and Methods, using a double-stranded oligonucleotide that corresponds to the −107/-83 position of the human Hsp70 promoter sequence (5′–CCC-CTG-GAA-TAT-TCC-CGA-CC–3′) containing an ideal HSE (in boldface). Two distinct HSE-binding proteins can be detected in this mobility shift assay: arrows indicate the HSF-1 slower migrating complex and the CHBF faster migrating complex. The autoradiography is representative of three independent experiments. Click here for file
