# Supplementary figures and images for: Heat shock protein 70 down-regulates the production of toll-like receptor-induced pro-inflammatory cytokines by a heat shock factor-1/constitutive heat shock element-binding factor-dependent mechanism
Source: J Inflamm (Lond). 2014 Jul 12;11:19. doi: 10.1186/1476-9255-11-19 (PMC4105516; doi:10.1186/1476-9255-11-19)

|                           |   |   |   |   |   |   |   |   |   |   |   |   |   |
|---------------------------|---|---|---|---|---|---|---|---|---|---|---|---|---|
| PGN                       | - | + | - | - | - | + | - | - | - | - | - | - | - |
| LPS                       | - | - | + | - | - | - | + | - | - | - | - | + | + |
| Hsp70                     | - | - | - | - | - | - | - | + | + | + | + | - | - |
| iTLR2                     | - | - | - | + | - | + | - | - | + | - | + | - | - |
| iTLR4                     | - | - | - | - | + | - | + | + | - | - | + | - | - |
| Cold NF- $\kappa$ B probe | - | - | - | - | - | - | - | - | - | - | - | + | - |
| Cold irrelevant probe     | - | - | - | - | - | - | - | - | - | - | - | - | + |

NF- $\kappa$ B

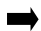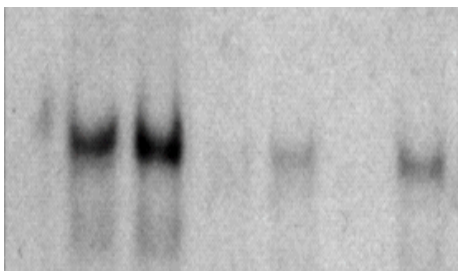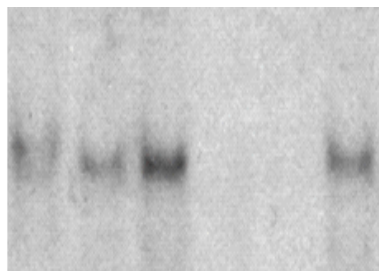

Supplement: Additional file 1: Figure S1 — Hsp70 activates NF-κB through TLR2 and TLR4 signaling. Peripheral blood mononuclear cells were blocked with 5 μg of anti-TLR2 antibody (iTLR2) and/or 30 μg of anti-TLR4 antibody (iTLR4). After 30 min, cells were washed and stimulated with 3 μg of Hsp70, 100 ng of LPS or 10 μg of peptidoglycan (PGN) for 120 min. After this incubation, nuclear extracts were obtained and analyzed by EMSA. The EMSA was performed as indicated in Material and Methods, using a double-stranded oligonucleotide that contains specific binding sites for NF-│B (5′–AGC-TAA-GGG-ACT-TTC-CGC-TGG-GGA-CTT-TCC-AGG–3′). Control included EMSA reaction with 50-fold excess cold NF-│B competitor or 50-fold excess irrelevant competitor. The autoradiography is representative of two independent experiments. The NF-κB complex is indicated (arrow). [file 1476-9255-11-19-S1.pdf]

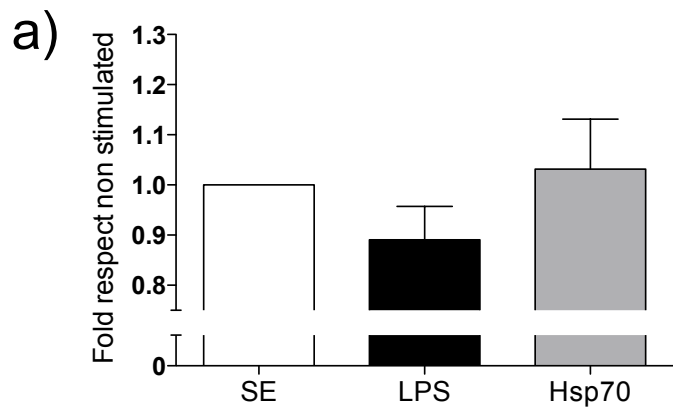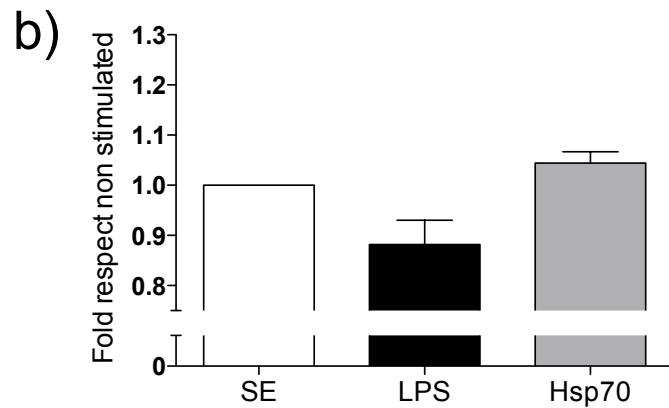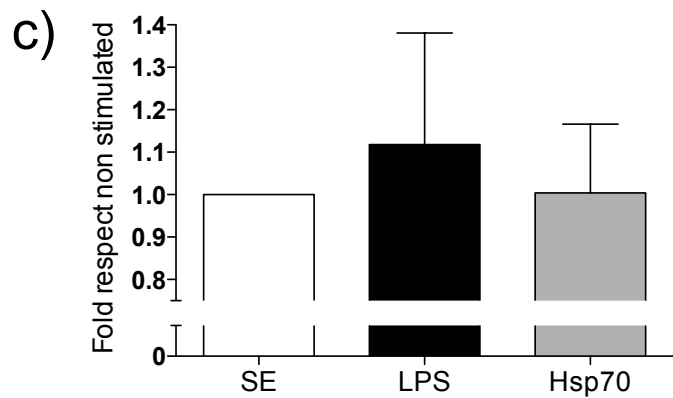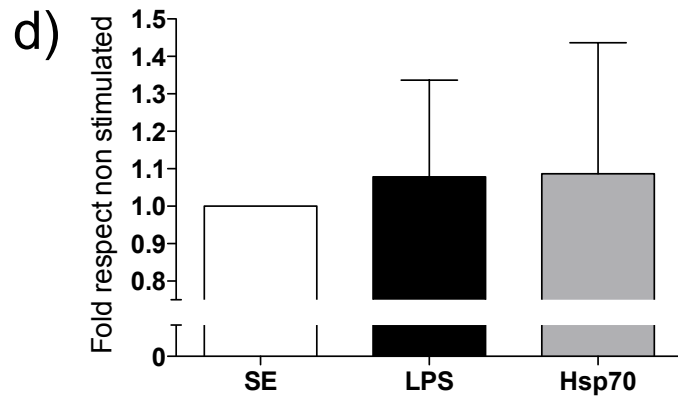

Supplement: Additional file 2: Figure S2 — Hsp70 has no effect on the surface expression of TLR2 and TLR4 on monocytes. Human peripheral blood mononuclear cells (5 × 10^5^) were stimulated with 3 μg/ml Hsp70 or 100 ng/ml of LPS (as a positive control of cellular activation) for 1 (a-b) or 4 hours (c-d). Surface expression of TLR2 (a-c) and TLR4 (b-d) in CD14+ cells was analyzed by flow cytometry after staining the cells with FITC-conjugated anti-TLR2, PE-conjugated anti-TLR4 and Pacific Blue-conjugated anti-CD14 (BioLegend) at room temperature for 30 min. TLR expression was normalized and is shown as fold expression, relative to untreated cells (NS). Data represent the mean ± SD of three independent experiments. [file 1476-9255-11-19-S2.pdf]

|                       |   |   |   |   |   |
|-----------------------|---|---|---|---|---|
| HS                    | - | + | - | - | - |
| LPS                   | - | - | + | + | + |
| Cold Hsp70 probe      | - | - | - | + | - |
| Cold irrelevant probe | - | - | - | - | + |

HSF1

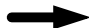

CHBF

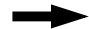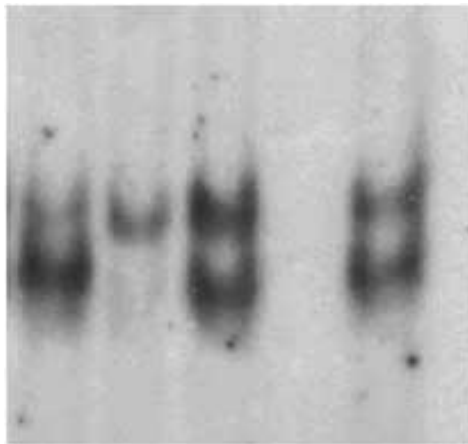

Supplement: Additional file 3: Figure S3 — Heat-shock induces HSF-1 binding to the human Hsp70 promoter. Peripheral blood mononuclear cells were incubated at 42°C for 1 h (heat-shock, HS), or stimulated with 100 ng/ml of LPS and incubated at 37°C for 1 h and nuclear extracts were obtained and analyzed by EMSA. The EMSA was performed as indicated in Material and Methods, using a double-stranded oligonucleotide that corresponds to the −107/-83 position of the human Hsp70 promoter sequence (5′–CCC-CTG-GAA-TAT-TCC-CGA-CC–3′) containing an ideal HSE (in boldface). Two distinct HSE-binding proteins can be detected in this mobility shift assay: arrows indicate the HSF-1 slower migrating complex and the CHBF faster migrating complex. The autoradiography is representative of three independent experiments. [file 1476-9255-11-19-S3.pdf]
